# Sketch of Burmah and Medical Science among the Burmese

**Published:** 1847-11

**Authors:** J. Dawson

**Affiliations:** Formerly of India


THE
### MEDICAL EXAMINER,
AND
### RECORD OF MEDICAL SCIENCE.

NEW SERIES.—No. XXXV.—NOVEMBER, 1847.

---

### ORIGINAL COMMUNICATIONS.

### *Sketch of Burmah and Medical Science among the Burmese.*

By J. Dawson, M. D., formerly of India.

Burmah presents to the eye of the traveller, the philanthropist, and man of science, diverse characteristics, which are as interesting as they are curious, and seem generally unknown to most of the enlightened nations of the earth. As an independent Asiatic sovereignty, with borders and boundaries tolerably well defined, it is a pretty considerable dominion, and is bounded on the north by China, on the south by Siam, on the east by the independent Shan states, and on the west by the territory and bay of Bengal. It is esteemed as being well supplied with a proper proportion of hills, mountains and vallies, small lakes, extensive rivers and springs, with plenty of excellent timber, and inexhaustible stores of geological and mineralogical productions. Vegetation is luxuriant, and both crops and fruits, when cultivated, are abundant. In a word, the country is rich in the possession of all the natural resources necessary for the support of an overflowing population. But from the peculiarity of the government, the nature of the climate and other causes, it contains nothing like the amount of people which it could decently maintain. The entire population, including Talines, Karens and Burmans, numbers about fifteen millions.

Its greatest geographical extent runs north and south, and is over 800 miles. Within this range of latitude, stretching some degrees beyond the tropic of Cancer, you have the extremes of wet and dry weather. To the north it is dry, the land in the neighbourhood of "Ummeerapoora"—the capital of the empire—being irrigated principally by periodical inundations of the Irrawaddie river. To the south it is wet, as much as 240 inches of rain falling during the year, 200 inches being the average quantity.

The government is a despotic monarchy, the King having complete control over the life, the liberty and property of any of his subjects. In his hands are the reins of absolute power, and he confides them to whom and how long he pleases. The name of the present sovereign of the kingdom is "Tharrawaddie."* He usurped the throne in 1837, and won the sceptre by deeds of blood. His several official titles are, His Golden-footed Majesty—King of the Rising Sun—Lord of the White Elephant, reigning over Thoo-na-pa-ran-ta, Tam-pa-dee-pa and many other great countries.

As a nation, the Burmese are usually classed among the half civilized races of the world. The people individually are humane, hospitable and destitute of bigotry, but as a nation they are proud, arrogant and self-sufficient. They have regular laws, both religious and civil. They have a literature and an established national faith. In their numerous books, called the *Beedaghat*, and *Demathat*, are contained the prescripts of their sacred and judicial institutions.

Gaudama is their divine lawgiver. He was their last incarnate deity, or Bhood. He died, it is said, of a diarrhœa, produced by eating pork. In some of their religious temples, he is represented as lying on a sick couch, with a doctor standing near him, having a pill between his fingers, and just ready to be administered as he fainted and expired. It is disrespectful to his memory, as well as positively sinful, to say that he is *dead*. Having entered "*Nike ban*," or the state of annihilation, he is regarded as no longer existing, either corporeally or spiritually. Believing in the transmigration of souls, of the passage of a spirit from one form or body into that of another, of the spirit of a man entering into that of an ox, a dog, or a cat, of a fish becoming a fowl, and a fowl a reptile, ultimate annihilation is the consummation of all their hopes and expectations in reference to eternity. During these almost endless peregrinations through the universe, in worlds unnumbered and unknown, the doctrine of personal merit is conspicuously held up to view. Do good and you will get good, is the old adage with them. Do evil and evil will recoil back upon your own head. By doing well they may be advanced to the dignity of angels and of gods. By doing ill they will be degraded to the level of fiends and fierce serpents.

* Intelligence has just reached of the death of this King.

Thus, the religion of the empire is Bhoodism. Unlike the Brahminism of the Hindoos, which is a cruel system of personal torture and bloody sacrifice, it is a humane system, based on certain laws, doctrines and precepts. It supports a priesthood and directs the building of magnificent temples and costly pagodas. It forbids the spilling of blood, even to the killing of a fowl, though monarchs have revelled in the slaughter of their unhappy victims. Contrasted with other forms of paganism and heathenish creeds, it might be considered as the mildest in principle, and the most humane operating system that could have originated in the mind of a mere man, unaided and unguided by any light drawn from the Christian religion. Some of their sacred laws are precisely analogous to those of the decalogue, recorded by Moses in the book of Exodus. Four of them are exactly similar in import, which prohibit killing, stealing, lying and adultery.

The institutes of Menu constitute their domestic and civil codes. Their criminal laws are very arbitrary. Decapitation is the common mode of executing malefactors; but on extraordinary occasions, emboweling, crucifixion with the head downwards, a horizontal division in a wedged position, by a saw into halves, tearing to pieces in quarters by elephants, have been cruelly and disgustingly practised.

Their books are not prepared of paper, but are formed of bundles of palm leaves, secured together upon a string. The writing is traced upon them by an iron point, stiletto, or pen.

The Burmese language appears to be derived from the "*Pali*," which is one of the ancient sacred languages of the East. Being composed chiefly of guttural, palatal and nasal sounds, the spoken language is not mellifluent, but full, difficult, and harsh to the ear of a European.

In stature, the people are short, muscular, and firmly built. In movements, the females are graceful. The men sometimes at particular feats are very energetic and sprightly, but ordinarily they are slow and inactive. Boat racing is a favourite exercise with them, and forms one of their great annual festivals. Few of them can be called stout or fat. The colour of their complexion varies from a light golden-yellow to that of a blackish-brown. The very darkest of them is much lighter coloured than a genuine African.

From the following particulars may be obtained a good idea of their physical proportions. It is an average of twenty-five men, who were examined for admission into the ranks of the Local Corps, serving in the Tennasserim provinces, under the jurisdiction of the English government.

Mean age of these individuals was twenty-four years.
" height, - - - five feet, three inches.
" weight, - - - eight stone two pounds.
Size round the chest, - - thirty-two inches.

Rice is emphatically the staff of life in India. Some condiment in the character of a curry is generally used with it. Many of the natives prefer a piece of roasted salt fish, with a sour vegetable, or fruit, to eat with the rice. They drink no coffee or milk, and very few Burmese take tea. Tea, however, is very frequently cooked up as a vegetable with garlic, onions, &c., and is prized as a delicacy. It is the customary offering when an application is made by a young man for the hand of a young lady in marriage.

The existing records of their ancient literature comprise many works on astronomy, astrology, metaphysics, history, chronology and medicine, beside some tables of the minor mathematics.

Medicine, as a science, is neither taught in schools, nor studied under private teachers. The profession has no medical colleges whatever in the country, and public hospitals are among the things which are yet to be founded. The people have none of the means or appliances possessed by enlightened nations for the acquisition and cultivation of this branch of useful learning.

The healing art is comprehended just about as well by the Burmese practitioners as it was by the bold, ignorant empirics of Greece, who practised before the time of Hippocrates. The most celebrated medical writer among them was *Zeewaka.* He was a disciple of Dee-tha-pa-mouk-ka, and flourished about 550 years anterior to the Christian era. To him are attributed many valuable treatises on medicine. His works, however, are nearly all lost, but his reputation is still cherished for his great learning and wisdom. For a knowledge of anatomy and mineral medicines he was eminently distinguished. Without striving to emulate his wisdom, his followers are content to revere him for his sagacity, admire him for his knowledge, and applaud him for his success. Since his days there have been many inferior writers, but none of any note within the period of modern times.

In questions of doubt or difficulty which may arise, it is a fundamental rule of the profession to refer everything to the authority of an ancient author. His opinion was so and so, and this is deemed enough. Possessing such a veneration for the ancients, and the fraternity apparently resting satisfied with what has hitherto been accomplished, has given rise to certain corruptions of these early writings. The practice of lauding to an extravagant degree these professional heirlooms, descending from previous ages, and, like the laws of the Medes and Persians, considering them unalterable, none having the moral courage of publicly advancing and vindicating any new theory that might occur to them, furnished some wily ingenious authors with opportunities of engrafting or adding their own supposed discoveries upon the original works of the old practitioners, whose writings, to the regret of all, have thus become garbled and filled with spurious passages.

Subjoined is an extract from a work which professes to be a treatise on anatomy, and claiming for itself a divine origin.

"The fœtus consists of thirty-two parts: its height becomes six feet; the hairs of the head are 25,000,000 in number; those of the body 99,099,000; the nails are 20; the teeth 32; the cuticle which covers the skin of the body (when rolled up) is the size of a cherry; the muscles are 900; the arteries 900; the veins 17,000; the bones 300; the joints 300; the bones are filled with marrow and sperm. The bones are distributed as follows: 9 in the upper skull, 1 in the forehead, 1 in the nose, 2 in the eyes, 2 in the ears, 2 in the jaws; the teeth are 32; 1 bone in the chin, 7 in the neck, 2 collar-bones, 2 shoulder-bones, 4 in the lower arms, 64 in the hands, 14 in the breast, 24 ribs, 1 in the middle of the lower breast, 18 in the back, 2 hip bones, 2 posterior bones, 4 thigh bones, (so in original,) 4 bones in the legs, 4 knee-bones, 4 bones in the heels, 64 in the feet, 64 soft smaller feet bones. There are also 100 more attached to the inner bones."

According to this arrangement of the osseous system, instead of 300, there are 434 distinct bones in the human body. If you were to point out the discrepancy between the two statements, it would only excite a smile. The mistake would remain unaltered and be perpetuated, because the physician could not say whether the one or the other was right, or whether both numbers might not be wrong.

Thus in anatomy all is ignorance and mystery. The mists and mazes of antiquity, with all its glorious uncertainties and boastful pretensions and authority, remain to be swept away. After this first step has been taken, then a lasting foundation might be laid from imported European and American medical science.

Chemistry is still a sealed casket of jewels, which possibly it will be the happiness of coming generations among them to see opened. Its beauties, even under the most common elementary forms, have yet to be unfolded and exhibited. An occasional pretender, however, has stepped on the platform of public notice, and declared with much vehemence that he has both seen and done wonders in the sublime and much vaunted science of alchemy. But like the philosophers of old, who have endeavoured to effect the transmutation of metals, and to discover a universal elixir for curing the whole round of human maladies, and for conferring longevity upon man, their names, their experiments and all, have justly been allowed to sink into the shades of a long-forgotten world of nonsense.

Of surgery they positively know nothing. Even the simple operation of phlebotomy they cannot, they dare not perform. When men have come begging to be bled, I have just inquired, why don't you go to your own doctors? The reply was: "*O! thu nama lay boo, theken.*" That is, *they know nothing about it, sir.*

Among the domestic customs of the Burmese is that of boring, at an early age, a hole through the lobe of each ear of both sexes. These holes are gradually enlarged by the introduction of successive pieces of thin twigs, till at length they become sufficiently large to admit a finger. In them, on particular occasions, gold ornaments are worn, or a bit of amber, glass, or polished marble. But the workmen or Coolies often insert their segars in them for convenience. Occasionally the lobe gets divided, or broken by some accident, when the man becomes filled with shame at his misfortune, or, if it be a woman, it is even worse. They will conceal it, as though it were the greatest deformity they could suffer. They will do anything in the way of trouble to have it united, but their own medical men are unequal to the task of helping them. In such cases, when they can, they apply to a foreign physician to join it by suture. Without flinching, they will sit to have it done; but if it were to perform any other operation, however trifling, except in a very few instances, the very sight of a lancet, scalpel, or pair of surgeon's scissors, would strike terror into them.

In old chronic swellings and tumours, they may, and sometimes do, in extreme cases, where there is much personal fortitude and courage, slightly scarify the part affected with a rude-pointed, rough-edged knife, or resort to actual cautery with a heated iron. At times, too, a few leeches, or a blister is applied. But they never, as a general thing, make more than one application of this nature.

Midwifery is practised by old women, the men having nothing to do with it, unless to give a dose of medicine when needed. The manual part certainly could not be monopolized by more dangerous hands. As a specimen of their treatment it may be mentioned, that when any sort of obstruction arises, the most cruel methods are pursued for overcoming it. From the roof of a house a long band is suspended, after the fashion of a swing, the female is placed upon it, her face looking to the floor, and urged with all her might to press downwards, to force away the child, her back being pressed at the same time with the knee or foot of one of her sweating attendants. If this does not prove effectual, she is stretched upon her back and is squeezed laterally along the sides by the hands of two old women, whilst a third places the heel of her foot on the abdomen, and thus makes pressure. Astringent and other washes are injected into the vagina with an instrument made of zinc and lead, resembling a miniature cannon, having rounded trunnions, by which it is grasped, and an ornamental piston rod. The sufferings of the poor injured woman no language can adequately portray. As might be expected, all such cases terminate in death. Happily they are not very numerous.

From time immemorial it has been the practice of these people to subject their women after parturition to a species of roasting for several days. Within the tropics, the heat at a certain season of the year is almost insufferable. To a high temperature is added a blazing fire, a few inches from which the parturient female is placed in a bed upon the floor, where she is kept for at least eight or ten days. After the birth of the child she is rubbed all over from head to feet with a stimulating yellow turmeric paste, to prevent her taking cold. The effect of the fire is said to excite secretion, and drain away all the noxious and impure fluids of the body.

Their notions of the development and growth of the fœtus are singularly crude, as displayed in the following quotation:

"At the first the fœtus is only water, not larger in the parent's womb than the drop detected with difficulty at the point of a hair of that sleek animal, the hyæna, after it has been immersed in oil clarified a hundred times, and from which the oil, so taken up, has been shaken one hundred times. The fœtus which is thus nothing but water, having become froth, (in this unchangeable way does it happen,) afterwards enters its third stage, and receives life and form; then the living fœtus becomes blended in its fourth stage; the two hands, the two feet and the head are produced and branch off into five extremities, the hair of the head and of the body, the ears, the eyes and the nose, are then formed."

The whole process is this: first, water; second, froth; third life and form; fourth, the branching, or growing out of the different members, as from a common centre which seems to be the trunk; and the fifth stage including the perfection of the head and the several limbs.

The periods of development here follow:

"The fœtus in its first stage of water is produced in fifteen days; its second stage is formed at the termination of one month; the third at the end of the second month; the fourth at the end of the third month; at the conclusion of four months, it branches forth into five extremities; at the fifth month the eyes and other members of sensation are formed. The fœtus remains ten months* (three hundred days) within the parent's womb, and after remaining there as in a repository, it comes forth."

*Two hundred and ninety-four according to others.

The Burmese say, that man is formed of the four great elements of "Earth," "Fire," "Air," and "Water."

"There are four elements of which the human frame is composed. The first is earth, the second, water, the next fire, the last, air; and in this manner are they distributed: there are twenty portions, or members formed of earth; viz.: the hair of the head, the hair of the body, the nails, the teeth, the cuticle of the skin, the flesh, the veins, the bones, the marrow of the bones, the lungs, the heart, the liver, the spleen, the stomach and the bowels, both large and small. Twenty-two parts are formed of water, gall, phlegm, pus, blood, sweat, indurated oil, tears, liquid oil, spittle, mucus, gluten, urine. Of fire, there are four parts: viz.: the embryo of fever, the principle of producing age, the embryo of melancholy, and the principle causing digestion. Of air there are six parts; the principle which occasions belching; that causing a breaking of wind; the embryo of disease in the stomach; the principle of circulation."

The practice of medicine seems to be followed only as an empirical art, and is mixed up with considerable superstition, sacred and vulgar. The following is an example of the rules which guide them.

"If a physician is called to attend upon a patient who lives to the south of his house, that person's disease cannot be healed (by him:) if the patient resides to the southwest, he can be cured in three days: if to the west, the complaint can also be gradually cured: if to the north, the patient will not completely recover: if called from the northeast upon Ta-nen-la (i. e. Monday,) he must not attend; if from the north, the sick person will not recover; if called from the east on a Monday and presented with a liberal fee before going, the patient will recover in three days, if the spirit be propitiated by an offering of food placed on the east side of the house. There is sometimes a pulse felt between the thumb and index finger of the right hand, at such times the subject is under the influence of witchcraft, the disease therefore is not a real one; boiled rice, fried fish and a fragrant condiment being placed on the north side of the house, as an offering to the spirit, the complaint will disappear. When there is a pulse in the middle joint of the right thumb, beware! it is the work of a household evil spirit to cause dejection of mind. When there is a throbbing pulsation in the fourth finger, the intellect of the person is obscured, and the body is parti-coloured; a witch and the household spirit possess the person. When the blood-watchers of life (the pulse in the wrist and foot) vibrate strongly, it is caused by an evil spirit: when they cease to beat, refrain from administering medicine—life is departing."

As the foregoing instructions to medical men are believed to be enjoined by divine authority, all reasoning as to the fitness or utility of the proposed cures is at once superseded. Their mystical influence, has become deep-rooted, from the acknowledged authority of accumulating ages, and though seemingly puzzling as a fact, that such a palpable chain of errors could have so long maintained its power, without a spirit to investigate, or a struggle to overthrow it, yet, unlike the gordian knot of the famous Phrygian chariot, it must be confessed that no Alexander has ever risen to cut it, and enfranchise the mind.

Every Burmese physician is strictly an empiric. Though some of them are conversant with a long catalogue of simples, still they have but one or two remedies for each disease, and when these fail, their skill is at an end. They appear unable understandingly to adapt means to ends. All is blind chance. In the use of the articles of the materia medica which they employ, they manifest but little judgment, and betray an imperfect acquaintance with their real virtues. Their manner of administering mercury, and other mineral medicines which enter into their materia medica, is open to the same objection.

In relation to Nosology, the whole circle of diseases is embraced under two grand classes. Their books of medical practice assert that "of the diseases to which the human frame is liable, there are ninety-six. Of which sixty-eight are wind complaints, and twenty-eight gall complaints. According to some others, there are eighty of the former, and sixteen of the latter." Symptomatology, which is the basis of good practice in any country, is but little understood. The meaning of symptoms, the difference between cause and effect, can hardly be intelligible, where there is such a notorious want of correct anatomical knowledge.

It is not a little amusing to witness the exploits of most of these warriors of physic. While a few of them appear to be deeply read in works on medicine, such as they are, and are otherwise generally intelligent, the mass are without a particle of book knowledge, and as ignorant almost as an untutored Indian. But there is this to be said in extenuation, that books are rather scarce with them, just as it was in Europe some two or three centuries ago, before the more general introduction of printing. A number, however, are collected together in some of the older Kyoungs, or monastries under the charge of the Bhoodist priests, where they are piled up for preservation as relics of the past, in large boxes, which are richly ornamented for this particular purpose.

The great field of medicine in Burmah, as in most other oriental countries, being entirely without any sort of barrier, or fence, in the shape of a legal test, or collegiate trial, every simpleton that pleases may ramble into its alluring precincts, to try his fortune. The only requisites for the office, are a brazen face, a flippant tongue, and a bag of drugs. If a Burman conceives to himself a desire to execute the functions of a doctor, he begins to practice at home. The first two or three cases are generally members of his own family; and having tried his ingenuity, he has perhaps been successful. As may be supposed this success is highly encouraging, and prompts him to march forward in his new career. The cases were probably such as would have turned out favourably without any kind of treatment. But this circumstance has nothing to do with it. In due time, after these astonishing triumphs have been achieved by our adventurer, friends in the neighbourhood call to make a visit. A story is got up relative to these wonderful cures that the new doctor has effected. Beating their breasts, the old folks look amazed and thunderstruck, and sing out, "*a mai, a mai,*" oh me, oh me, how wonderful! Assuming an air, of dignity and an apparant earnestness, the "*Sa tha mah,** *theken,*" for such now becomes his title, attempts to explain the nature of the disease, and above all how easily it could be managed in his hands and by his medicine. All this great matter is of course borne in mind by the visitors. Shortly after the doctor receives a call to see a sick person residing at the other end of the town. If he happens to think that the fates are not against him, he goes, but should the time be unpropitious, he declines and waits for another patient.

Having thus entered the professional sphere, the gentleman has to bestir himself, to look dignified, and furnished with drugs like a regular master of the healing art. A bag to throw over the shoulder is provided. This is to carry with him all the medicines he requires when going to visit the sick. His stock, which is contained in the cotton bag, mostly consists of a little brimstone and some bluestone, some mercury and crude antimony, a small lump of opium, a few bitter and aromatic roots, some powdered spices, as cloves, cinnamon, black pepper and ginger, either mixed or separate in small tin boxes, a little garlic and capsicum, a hog's tooth or two, or a couple of teeth of some wild animal, the claws or nails of a tiger, a flat piece or two of metal, some cotton and woolen strings or thread of different colours, (all the latter are to apply as charms,) and a China cup or two for giving medicine. In the majority of instances this enumeration comprises the whole drug establishment of the native doctors of that country. There is, however, a class of druggists to be found in the public bazaars, who keep a larger and more varied stock, and sell to the practising physicians and quacks.

When summoned to visit a patient, the practitioner makes the person sent sit down and give some account of the case. Having thus learned a few of the particulars, a fee is demanded in advance to buy medicines. A message is then returned to say that the physician will be in attendance presently with his remedy. Reaching the house, he steps in, and walks right up to the sick man. There is no ceremony of bowing and scraping observed,

* "*Sa tha mah*" literally means doctor, or master of medicine. "*Theken*" is a term of respect, and signifies friend, sir, master, or lord.

as may be seen in some more civilized parts, and no compliments are exchanged of wishing "*good day*," &c. The doctor sits down on the floor, by the side of the individual, and proceeds to feel the pulse, looks into the eyes, nose and ears, and sometimes into the mouth. Whether the mouth is omitted in the examination intentionally, or from neglect, I have not heard. But as the whole nation, without exception, chew the beetle leaf and nut, with slacked lime, tobacco and catechu all mixed, it keeps the mouth constantly stained of a red colour. Crusts form on the teeth from this habit. Very seldom a question is asked in reference to pains, aches, &c. The esculapian is supposed, or pretends to know all about them from the pulse. The wonderful pulse, according to his theory, tells everything, and in all certainty he understands nothing whatever of the circulation, and but little of the internal structure of the human economy. How can he, it may be inquired, for he has *never seen* the inside of a dead body, i. e. the person of a man, or woman.

The amount of their written information, (and this as we have already seen, is known only to a few of them,) respecting the several organs, is exhibited in the extract given below, taken from a book on anatomy, there being no work extant on the specific subject of physiology.

"The heart in shape is like the bud of a water lily; it lies with the point downwards, having three folds at the lower end, and is situated in the middle of the breast; it contains about a handful of blood."

"The liver of a *wise man* is thin and divided into three lobes: the unwise have large deformed livers: the liver is on the right side under the heart."

"The first spleen is in contact with the bowels; it is eight fingers in length, and in shape is like a cow's tongue. The second spleen is on the right side of the liver."

"There are three kinds of lungs, red, black, and spotted; they are situated in the breast above the liver, and are in shape like the fruit of the "*thapan*," (mirobalans.)

"The intestines of a man are thirty-two cubits long, those of a female twenty-eight cubits; they are coiled up in twenty-one folds below the opening of the breast."

"There are two descriptions of gall; one is contained in a bag, in contact with the liver; the other is diffused through the whole body."

"The human frame is also filled with pus."

"Perspiration exists in the orifices from which the hair grows."

"The spittle flows from the pouches on each side of the jaws."

Here is a specimen of jumbling of the most finished touch. The liver is again honored by having allotted to it the lodging place of wisdom. The first spleen must be the pancreas, but how the spleen itself could be assigned to the right side it is not easy to conjecture. The urinary organs seem to be entirely overlooked in this description.

But we must follow the doctor in his practice. Shampooing is a favorite auxiliary in the treatment of disease. It is put into operation at once. The physician himself squeezes away in every direction; hands, arms and shoulders, feet, legs and thighs, as well as the trunk, all get a share of attention. A dose is then administered to the patient, mixtures being more commonly given than pills. The nostrils are then plied well with some aromatic powder. Next, a charm is formally applied to the neck, wrists or ankles. Certain offerings are proposed to be made to the "*nats*," or evil spirits. And whether fever or inflammation be present, exhaustion or languor, or anything else, it matters not what, the patient must be stuffed, as a part of the treatment, with quantities of boiled rice. It is given in boluses the size of a peach. The doctor, looking very grave and stern, informs the sick person and his friends, that "*unless he eats he must surely die.*" The whole proceeding, from first to last, would be regarded by a lover of the stage, as a beautiful farce, divested of all appearance of a plot, but full of action and droll amusement.

The treatment pursued is highly stimulating. Emetics and cathartics are very rarely used. There is a horror of having the bowels moved freely. When the natives seek the aid of a foreign practitioner, they often inquire whether the dose he gives will purge. Frequently the dose has been handed back, when told that it would, with a very significant nod of the head and the exclamation, "*Ah! ma kom boo,*" which means, "*Ah! that's very bad.*" Their last deity having been carried off in consequence of a looseness, seems to awaken in them a strong suspicion in relation to the fatal nature of free abdominal evacuation. In addition to this historical circumstance, their own purgatives are of the most drastic, violent character, and being oftener administered without, than with, either judgment or caution, is perhaps a sufficient cause to create a general dislike to them among the people. Though this is the popular opinion of the present age, it was not the universal rule of antiquity, as is manifest in some of the old treatises on practical medicine still extant, in which they figure in common with other things. In combination with a number of stimulating ingredients, their formula for certain compound preparations occasionally display the presence of some active cathartic article.

A composition of this sort is presented in the following recipe. The prescription as recorded, is directed to be employed in bowel complaints and pains in the abdomen. It betrays the exciting nature of the remedies, to which they were and are still accustomed to resort.

| | | |
|---|---|---|
| "Capsicum, - - - - | (Bird-eye pepper) fruit, | (quantity illegible.) |
| Piper longum, - - - - - - - | " - | 100 grains. |
| Amomum Zingiber, - - - - - - - - | root | 100 " |
| Allium Sativum, - - - - - - - - | " | 100 " |

| | | | |
|---|---|---|---|
| Anethum fœniculum, - - - - - - - | seed | 100 | grains. |
| Croton Tiglium, - - - - - - - - | " | 10 | " |
| Gentiana Chirayita, - - - - - - - - | root | 10 | " |
| Myristica, - - - - - - - - - | fruit | 10 | " |

"A quantity of Cactus triangularis to be sliced, the juice drawn from it and thrown away. The remaining part should be added to the above, and both well triturated together. To be taken in quantities of ——* grains when required. Previous to taking this medicine, it must be dipped in salt."

Besides the efforts of the regular practitioners thus put forth in behalf of the sick, the agency of quacks and silly old women is sought, when the illness of a person is supposed to depend on mischief perpetrated by imps, wizards, hobgoblins and evil spirits. All kinds of mental alienation and disorder are laid at their door, as likewise diseases of a lingering character. The women thus called upon to exercise their craft, for the removal of these imaginary phantoms and spirits, would remind one very forcibly of the poor creatures described by writers on "Demonology," that were burnt as witches and wizards in Scotland and England, during the reign in those countries of superstition, religious intolerance and persecution.

The ceremony for dislodging these pernicious invisibles is somewhat ludicrous. A sort of shrine, with silver and marble images, leaves and flowers, is erected on a raised platform of bamboos. Before it are deposited the different offerings, in cups and on plates. An elderly female is dressed up fantastically, in tattered garments of singular shapes and colours, and decked out with wreaths of flowers and feathers. She holds in each hand a long sword; and in front of the shrine, cutting all manner of antics and capers, she dances to music. This mystical ceremony is sometimes prolonged through the period of a whole day and night, or a couple of days, by the appearance now and then of the female referred to, and of a succession of comical fellows, who dance in the same way, and kick up no uncommon kind of shindy. By looking at them, one is irresistibly led to exclaim, O what stupid infatuation! What worthless folly of concerted phrenzy and vain hope!

To judge from what an observer will witness, relative to the feeling entertained by the people toward their own medical men, the conclusion is, that they do not appear to have any great confidence in their skill, or modes of practice. The Burmese are indeed very fickle in this respect. They somewhat unreasonably desire to see *speedy* results for good, follow the employment of medicinal means. They soon become discouraged, and quickly relinquish hope, if such do not turn up. At times as many as a dozen practitioners may be called in attendance on a case, in the

*The quantity directed to be given is not legible in the original.

course of a single day, and dismissed one after another, as soon as each one has administered his dose. If the patient die while the last physician is in the house, *he gets the name of having done the business.* His predicament then is anything but agreeable or pleasant. The poor fellow sneaks out, amid the noise and lamentations that are raised, as if he had by some unhappy blunder really committed a murder.

The Burmese burn their dead. Young children alone are buried. Ordinary funerals are solemnly grand. In burning a priest there is great display made. In speaking of the decease of aged persons, they compare it by a figure of speech which they use, "*to the dropping of ripe fruit from a tree.*"

Here I must close the subject, not because it is exhausted, but from an apprehension of wearying my readers. Imperfect and rambling as this sketch may seem, an inquirer can gather from it *some* of the more prominent facts connected with the present state of Medicine in that interesting kingdom of Asia.

NOTE. The translation from Burmese into English of the several passages quoted in this paper, was made by a gentleman distinguished for his acquirements and knowledge of the literature of that empire.

